# Generalized Structured Component Analysis with Uniqueness Terms for Accommodating Measurement Error

**DOI:** 10.3389/fpsyg.2017.02137

**Published:** 2017-12-06

**Authors:** Heungsun Hwang, Yoshio Takane, Kwanghee Jung

**Affiliations:** ^1^Department of Psychology, McGill University, Montreal, QC, Canada; ^2^Department of Psychology, University of Victoria, BC, Canada; ^3^Department of Educational Psychology and Leadership, Texas Tech University, Lubbock, TX, United States

**Keywords:** generalized structured component analysis, uniqueness, measurement error, bias correction, structural equation modeling

## Abstract

Generalized structured component analysis (GSCA) is a component-based approach to structural equation modeling (SEM), where latent variables are approximated by weighted composites of indicators. It has no formal mechanism to incorporate errors in indicators, which in turn renders components prone to the errors as well. We propose to extend GSCA to account for errors in indicators explicitly. This extension, called GSCA_M_, considers both common and unique parts of indicators, as postulated in common factor analysis, and estimates a weighted composite of indicators with their unique parts removed. Adding such unique parts or uniqueness terms serves to account for measurement errors in indicators in a manner similar to common factor analysis. Simulation studies are conducted to compare parameter recovery of GSCA_M_ and existing methods. These methods are also applied to fit a substantively well-established model to real data.

## Introduction

Structural equation modeling (SEM) involves the specification and testing of the relationships between variables that are observed (indicators) and unobserved (latent variables). Two approaches have been proposed for SEM: Factor-based vs. component-based (e.g., Fornell and Bookstein, 1982; Jöreskog and Wold, 1982; Tenenhaus, 2008; Rigdon, 2012). The former includes covariance structure analysis (CSA; Jöreskog, 1970, 1973), and the latter includes partial least squares path modeling (PLSPM; Wold, 1966, 1973, 1982; Lohmöller, 1989) and generalized structured component analysis (GSCA; Hwang and Takane, 2004, 2014). As their names imply, the two approaches become divergent in how they approximate latent variables in these sub-models. That is, factor-based SEM assumes that common factors may approximate latent variables as in common factor analysis, whereas component-based SEM posits that weighted composites of indicators may serve as proxies for latent variables as in principal component analysis. In this regard, the two approaches are conceptually different, and choices on them would likely be application-dependent and should be theoretically decided in advance, considering how to conceptualize latent variables in the application as well as how to validate the relation between latent variables and their proxies (factors or components; Rigdon, 2012).

Nonetheless, in comparison with factor-based SEM, perhaps the most common criticism of component-based SEM has been that it has no mechanism to formally take into account errors in indicators, which appear practically inevitable in the social sciences (e.g., Bentler and Huang, 2014). This also leads components to take over these errors to some extent, although extracting a weighted composite from a set of indicators can play a role in reducing the errors implicitly (Gleason, 1973). It is well-known that errors in independent (observed or latent) variables will likely result in biased parameter estimates (e.g., Bollen, 1989, Chapter 5).

To deal with this problem, a bias-correction method, called consistent partial least squares (PLSc; Dijkstra, 2010; Dijkstra and Henseler, 2015), has been proposed in the context of PLSPM. The basic premise of PLSc is that the true measurement model is the so-called basic design (Wold, 1982), representing a unidimensional confirmatory factor analytic model, where each latent variable/factor underlies at least two indicators with each indicator loading on one and only one latent variable. Under this assumption, PLSc begins to apply PLSPM to estimate component weights for a set of indicators per latent variable and then obtains a correction constant for the set of indicators based on their component weights and sample correlations. The loadings for the indicators are estimated by multiplying their component weight estimates by the correction constant. The correlations among latent variables are also estimated using the correction constants for all sets of indicators, which are subsequently used for estimating path coefficients. Conceptually, it is somewhat arbitrary whether PLSc falls into component-based SEM because it has little interest in the specification and estimation of components *per se*, and simply utilizes their weight estimates to obtain parameter estimates of factor-based SEM. In practice, the assumption of the basic design can be restrictive, leading to the exclusion of cross loadings that have been well-accepted in numerous structural equation models (Asparouhov and Muthén, 2009). For example, a classical model involving cross loadings is a multitrait-multimethod model, where trait and method latent variables underlie each indicator (Campbell and Fiske, 1959). Another example is latent growth curve models (Meredith and Tisak, 1990; Duncan et al., 2006), where indicators are typically assumed to load on multiple latent variables, each of which captures a different trajectory of change over time.

To our knowledge, no attempts have been made to incorporate errors in indicators or develop a bias-correction strategy in the context of GSCA. Thus, in this paper, we propose to extend GSCA to explicitly account for errors in indicators. Specifically, we aim to combine a unique part of each indicator into GSCA. As postulated in common factor analysis or factor-based SEM, adding such a unique part may be seen as accounting for measurement error in each indicator. We shall call this proposed extension “GSCA_M_,” standing for GSCA with measurement errors incorporated. GSCA_M_ will provide parameter estimates comparable to those from factor-based SEM. Whereas PLSc involves two separate estimation steps, GSCA_M_ has a single estimation procedure where a least squares criterion is consistently minimized to estimate all model parameters. In addition, GSCA_M_ does not require the basic design assumption in model specification and parameter estimation.

The paper is organized as follows. Section Method discusses the technical underpinnings of GSCA_M_, including model specification and parameter estimation. Section Simulation Studies conducts a simulation study to evaluate the performance of GSCA_M_ and two existing methods—CSA and PLSc. Section An Empirical Application presents an application to show the empirical usefulness of GSCA_M_ as compared to the existing methods. The final section summarizes the implications of the proposed method.

## Method

### Model

As with GSCA, GSCA_M_ involves three sub-models—measurement, structural, and weighted relation. The measurement model is used to specify the relationships between indicators and latent variables, whereas the structural model is to specify the relationships among latent variables. The weighted relation model is used to express a latent variable as a weighted composite of indicators. Unlike GSCA, however, GSCA_M_ contemplates both common and unique parts of each indicator in the measurement model, and expresses a latent variable as a weighted composite of indicators with their unique parts removed in the weighted relation model.

Let **Z** = [**z**_1_,…, **z**_*J*_] denote an *N* by *J* matrix of indicators, where *N* is the number of observations, and **z**_*j*_ is the *j*th indicator (*j* = 1,…, *J*). Let **Γ** = [**γ**_1_,…, **γ**_*P*_] denote an *N* by *P* matrix of latent variables, where **γ**_*p*_ is the *p*th latent variable (*p* = 1,…, *P*). Assume that all indicators and latent variables are normalized such that their lengths are equal to one (i.e., $z_{j}\prime z_{j}=\gamma_{p}\prime \gamma_{p}=1$). Let **C** denote a *P* by *J* matrix of loadings relating latent variables to indicators. Let **U** denote an *N* by *J* matrix of unique variables. Let **D** denote a *J* by *J* diagonal matrix of unique loadings. Let **E**_1_ denote an *N* by *J* matrix of residuals for indicators. Let **B** denote a *P* by *P* matrix of path coefficients connecting latent variables among themselves, and **E**_2_ denote an *N* by *P* matrix of residuals for latent variables. The three sub-models of GSCA_M_ are given as follows.

(1) $$Z=\Gamma C+UD+E_{1}$$

(2) $$\Gamma =\Gamma B+E_{2}$$

(3) Γ=(Z−UD)W.

In the measurement model (1), **ΓC** and **UD** represent common and unique parts of indicators, respectively. We assume that **Γ** is uncorrelated with **U** (**U**′**Γ** = **Γ**′**U** = **0**) and **U** is orthonormalized ($U\left(U^{\prime }\Gamma =\Gamma^{\prime }U=0\right)$, where **I**_*J*_ is the identity matrix of order *J*). In the measurement model, the **C** matrix contains fixed values (e.g., zeros) to accommodate hypothesized relationships between indicators and their latent variables as in confirmatory factor analytic models. If this matrix has no fixed values, (1) may be seen as the fixed exploratory factor analytic model (Young, 1941; de Leeuw, 2004, 2008). The structural model (2) remains the same as that for GSCA or the reticular action model (McArdle and McDonald, 1984). The weighted relation model (3) shows that a latent variable is defined as a weighted composite of indicators with their unique parts eliminated.

GSCA_M_ integrates the sub-models into a single equation, as follows.

(4) $$\begin{array}{l} \left[Z,\Gamma ]=\Gamma [C,B]+[UD,0]+[E_{1},E_{2}\right]\\ \;\Psi =\Gamma A+S+E, \end{array}$$

where **Ψ** = [**Z**, **Γ**], **A** = [**C**, **B**], **S** = [**UD**, **0**], and **E** = [**E**_1_, **E**_2_]. This is called the GSCA_M_ model.

### Parameter estimation

The parameters of GSCA_M_ (**Γ**, **A**, **U**, and **D**) are estimated by minimizing the following least squares criterion

(5) ϕ=SS(Ψ−ΓA−S),

subject to $\gamma_{p}^{\prime }\gamma_{p}=1$or equivalently diag(**Γ**′**Γ**) = **I**_*P*_, **U**′**Γ** = **0**, and $\left(\Gamma^{\prime }\Gamma \right)=I_{P},U^{\prime }\Gamma =0$, where SS(**X**) = tr(**X**′**X**), and **I**_*P*_ is the identity matrix of order *P*.

A simple iterative algorithm is developed to minimize (5). This algorithm begins by assigning initial values to the parameters. Then, it alternates several steps until convergence, each of which updates a set of parameters in a least squares sense, with the other sets fixed. A detailed description of the algorithm is provided in the Appendix in Supplementary Material.

We can apply GSCA to obtain initial values for **Γ**, **C**, and **B**, although any other initial values can be considered. Then, those for **U** and **D** may be obtained as described in Steps 3 and 4 in the Appendix in Supplementary Material. We can employ the bootstrap method (Efron, 1979) to estimate the standard errors or confidence intervals of the parameter estimates without resorting to a distributional assumption such as multivariate normality of indicators. When the number of observations is smaller than that of indicators (*N* < *J*), rank(**U**) < *J* and the constraint $U^{\prime }U=I_{J}$ cannot be fulfilled. In this situation, we may apply Unkel and Trendafilov's (2013) algorithm to update **U**, subject to the new constraint **U**′**UD** = **D** (also see Trendafilov and Unkel, 2011). We assume that all indicators and latent variables are normalized, which still results in standardized parameter estimates except for latent variable scores that are normalized. The standardized latent variable scores are obtained by multiplying the normalized scores by $\sqrt{N}$.

GSCA_M_ can provide a measure of overall model fit, called FIT. The FIT indicates the total variance of all variables explained by a particular model specification. It is given by

(6) $$\text{FIT}=1-\frac{\text{SS}(\Psi -\Gamma A-S)}{\text{SS}(\Psi )}$$

The values of the FIT range from 0 to 1. The larger this value, the more variance in the variables is accounted for by the specified model. Moreover, it can provide separate model fit measures for the measurement and structural models, as follows.

(7) $${\text{FIT}}_{\text{M}}=1-\frac{\text{SS}(Z-\Gamma C-UD)}{\text{SS}(Z)},$$

(8) $${\text{FIT}}_{\text{S}}=1-\frac{\text{SS}(\Gamma -\Gamma B)}{\text{SS}(\Gamma )}$$

The FIT_M_ shows how much the variance of indicators is explained by a measurement model, whereas the FIT_S_ indicates how much the variance of latent variables is accounted for by a structural model. Both measures range from 0 to 1 and can be interpreted in a manner similar to the FIT.

## Simulation studies

We conducted simulation studies to evaluate the performance of GSCA_M_ as compared to existing methods, including GSCA, PLSc, and CSA. In particular, we focused on comparing GSCA_M_ to these methods in parameter recovery.

### Simulation study 1

**Figure 1:**
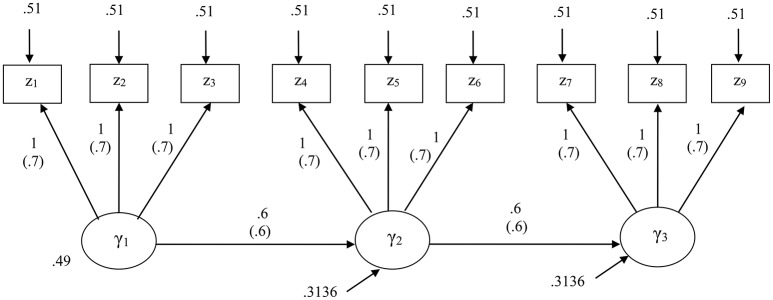
The structural equation model specified for the first simulation study. Standardized parameters are given in parentheses.

We specified a structural equation model that consisted of three latent variables and three indicators per latent variable. Figure 1 displays the specified model along with their unstandardized and standardized parameter values.

For this study, we considered four levels of sample size: *N* = 100, 200, 500, and 1,000. At each sample size, we generated 1,000 random samples from a multivariate normal distribution with zero means and the covariance matrix implied by the unstandardized parameters of the correct model, based on a CSA formulation. We used the maximum likelihood method for CSA. For PLSc, we used Mode A and the path weighting scheme that is preferred over the other schemes (centroid and factorial) in estimating component weights (Esposito Vinzi et al., 2010). We used the same random seed for all the methods for each sample to have them run with the same initial values.

Table 1 exhibits the relative biases (expressed as percentages), standard deviations, and mean square errors of the standardized loadings and path coefficients estimated from the four methods over the different sample sizes. In the calculations of these properties, we removed any sample involving non-convergence within 300 iterations or convergence to improper solutions. CSA encountered such convergence problems across all the sample sizes. The number of the samples omitted with the convergence problems under CSA was as follows: 66 (*N* = 100), 46 (*N* = 200), 31 (*N* = 500), and 31 (*N* = 1,000). PLSc was faced with the problems only when *N* ≤ 200. Specifically, 23 and 2 samples were omitted when *N* = 100 and 200, respectively. Conversely, GSCA and GSCA_M_ did not encounter non-convergence or the occurrence of improper solutions across all the sample sizes.

**Table 1:** Relative biases expressed as percentages (RB(%)), standard deviations (SD), and mean square errors (MSE) of standardized loadings and path coefficients obtained from GSCA, GSCA_M_, PLSc, and CSA over different sample sizes.

***N***	**Parameters**		**RB (%)**				**SD**				**MSE**			
			**GSCA**	**GSCA_M_**	**PLSc**	**CSA**	**GSCA**	**GSCA_M_**	**PLSc**	**CSA**	**GSCA**	**GSCA_M_**	**PLSc**	**CSA**
100	Loadings	0.7	15.77	0.64	−1.11	−0.03	0.04	0.09	0.12	0.08	0.01	0.01	0.01	0.01
		0.7	15.91	1.04	−0.79	−0.14	0.04	0.09	0.11	0.08	0.01	0.01	0.01	0.01
		0.7	15.59	0.33	−1.29	−0.73	0.04	0.09	0.11	0.08	0.01	0.01	0.01	0.01
		0.7	16.00	1.49	−0.33	0.37	0.04	0.09	0.09	0.08	0.01	0.01	0.01	0.01
		0.7	15.80	0.56	−0.09	−0.29	0.04	0.09	0.09	0.07	0.01	0.01	0.01	0.01
		0.7	15.60	1.04	−1.07	−0.54	0.04	0.09	0.09	0.08	0.01	0.01	0.01	0.01
		0.7	15.80	0.34	−0.50	−0.46	0.04	0.09	0.11	0.08	0.01	0.01	0.01	0.01
		0.7	15.97	1.44	−1.19	0.26	0.04	0.09	0.12	0.08	0.01	0.01	0.01	0.01
		0.7	15.93	0.91	−0.64	0.03	0.04	0.09	0.11	0.08	0.01	0.01	0.01	0.01
	Paths	0.6	−25.00	−4.65	0.87	−0.60	0.08	0.10	0.10	0.11	0.03	0.01	0.01	0.01
		0.6	−24.52	−4.08	1.42	−0.02	0.08	0.10	0.10	0.10	0.03	0.01	0.01	0.01
200	Loadings	0.7	15.80	−0.01	−0.36	−0.43	0.03	0.06	0.08	0.05	0.01	0.00	0.01	0.00
		0.7	15.90	0.27	−0.51	−0.10	0.03	0.06	0.08	0.05	0.01	0.00	0.01	0.00
		0.7	16.06	0.80	−0.41	0.17	0.03	0.06	0.08	0.05	0.01	0.00	0.01	0.00
		0.7	15.94	0.24	0.01	−0.07	0.03	0.06	0.08	0.05	0.01	0.00	0.00	0.00
		0.7	15.94	0.60	−0.33	−0.17	0.03	0.06	0.08	0.05	0.01	0.00	0.00	0.00
		0.7	15.99	0.71	−0.31	−0.06	0.03	0.06	0.08	0.05	0.01	0.00	0.00	0.00
		0.7	16.00	0.39	−0.40	0.01	0.03	0.06	0.08	0.05	0.01	0.00	0.01	0.00
		0.7	15.96	0.50	−0.33	−0.03	0.03	0.06	0.08	0.05	0.01	0.00	0.01	0.00
		0.7	15.97	0.47	−0.36	0.01	0.03	0.06	0.08	0.05	0.01	0.00	0.01	0.00
	Paths	0.6	−25.10	−1.82	0.67	−0.18	0.06	0.07	0.07	0.07	0.03	0.01	0.01	0.01
		0.6	−24.90	−1.63	0.87	0.18	0.06	0.07	0.07	0.07	0.03	0.01	0.01	0.00
500	Loadings	0.7	16.03	0.14	−0.03	0.00	0.02	0.04	0.05	0.03	0.01	0.00	0.00	0.00
		0.7	16.01	0.20	−0.30	−0.13	0.02	0.04	0.05	0.03	0.01	0.00	0.00	0.00
		0.7	16.09	0.29	0.01	0.10	0.02	0.04	0.05	0.03	0.01	0.00	0.00	0.00
		0.7	16.06	0.33	−0.09	0.06	0.02	0.04	0.04	0.03	0.01	0.00	0.00	0.00
		0.7	15.96	0.11	−0.19	−0.11	0.02	0.04	0.04	0.03	0.01	0.00	0.00	0.00
		0.7	16.00	0.09	−0.04	−0.06	0.02	0.04	0.04	0.03	0.01	0.00	0.00	0.00
		0.7	16.03	0.17	−0.13	−0.14	0.02	0.04	0.05	0.04	0.01	0.00	0.00	0.00
		0.7	16.07	0.37	−0.34	−0.11	0.02	0.04	0.05	0.06	0.01	0.00	0.00	0.00
		0.7	16.04	0.07	0.21	−0.23	0.02	0.04	0.05	0.06	0.01	0.00	0.00	0.00
	Paths	0.6	−25.73	−1.02	−0.10	−0.35	0.04	0.04	0.05	0.04	0.03	0.00	0.00	0.00
		0.6	−25.32	−0.45	−0.45	−0.02	0.04	0.05	0.05	0.06	0.02	0.00	0.00	0.00
1,000	Loadings	0.7	16.04	0.13	−0.13	0.00	0.01	0.03	0.04	0.02	0.01	0.00	0.00	0.00
		0.7	16.01	0.04	−0.14	−0.13	0.01	0.03	0.03	0.03	0.01	0.00	0.00	0.00
		0.7	16.06	0.07	0.07	0.01	0.01	0.03	0.04	0.02	0.01	0.00	0.00	0.00
		0.7	16.01	0.01	−0.04	−0.03	0.01	0.03	0.03	0.02	0.01	0.00	0.00	0.00
		0.7	15.94	−0.10	−0.17	−0.13	0.01	0.03	0.03	0.02	0.01	0.00	0.00	0.00
		0.7	15.97	0.07	−0.21	−0.11	0.01	0.03	0.03	0.02	0.01	0.00	0.00	0.00
		0.7	16.03	−0.04	0.04	−0.19	0.01	0.03	0.04	0.03	0.01	0.00	0.00	0.00
		0.7	16.14	0.30	0.09	0.20	0.01	0.03	0.03	0.02	0.01	0.00	0.00	0.00
		0.7	16.07	0.23	−0.09	0.11	0.01	0.03	0.03	0.02	0.01	0.00	0.00	0.00
	Paths	0.6	−25.60	−0.25	0.20	0.15	0.02	0.03	0.03	0.03	0.02	0.00	0.00	0.00
		0.6	−25.67	−0.40	0.07	0.02	0.03	0.03	0.03	0.03	0.02	0.00	0.00	0.00

*A factor-based structural equation model with equal loadings was specified*.

We regarded relative bias >10% in absolute value as indicative of an unacceptable degree of bias (e.g., Bollen et al., 2007; Lei and Wu, 2012). As shown in Table 1, GSCA provided positively biased loading estimates and negatively biased path coefficient estimates across all the sample sizes. This is expected because the simulated data were generated based on a CSA formulation, assuming that a latent variable was equivalent to a common factor. In this case, component-based approaches to SEM, such as GSCA and PLSPM, are known to overestimate loadings and underestimate path coefficients (e.g., Velicer and Jackson, 1990; Dijkstra, 2010; Hwang et al., 2010; Sarstedt et al., 2016). Conversely, GSCA_M_, PLSc, and CSA tended to result in unbiased estimates of both loadings and path coefficients across the sample sizes. When *N* = 100, however, the path coefficient estimates of GSCA_M_ showed larger relative biases (4–5%) than those from PLSc and CSA, although they decreased rapidly with the sample size, approaching essentially zero when *N* = 1,000.

The standard deviations of the estimates from the four methods became smaller with the sample size. However, GSCA provided smaller standard deviations than the other methods. This was particularly salient when the sample size was small (e.g., *N* = 100), which is consistent with the literature (Hwang et al., 2010). When the sample size was small, all the methods tended to show similar mean square errors of the loading estimates, whereas GSCA tended to provide larger mean square errors of the path coefficient estimates. As the sample size increased, the mean square errors of all the parameter estimates obtained from GSCA_M_, PLSc, and CSA approached zero, while those from GSCA remained slightly larger.

### Simulation study 2

**Figure 2:**
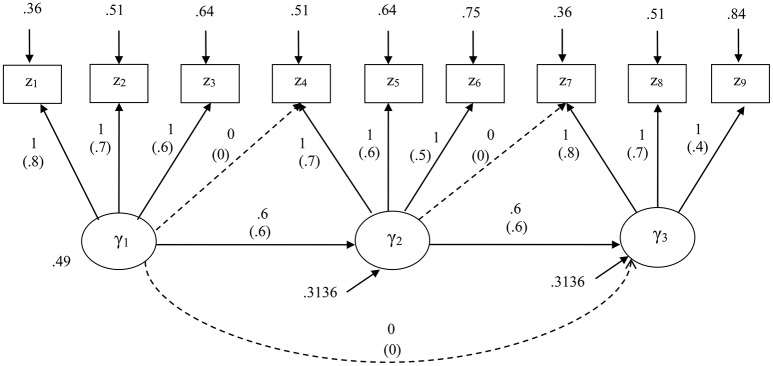
The structural equation model specified for the second simulation study. Standardized parameters are given in parentheses. A model misspecification involves addition of two cross loadings and a path coefficient as indicated by dashed arrows.

The first simulation study was useful to evaluate how GSCA_M_ performed as compared to different methods. Nonetheless, this study considered a model with equal loadings, which might be too ideal in reality. Thus, we conducted another simulation study to compare the performance of the methods under a model with the same structure but unequal loadings varying from 0.4 to 0.8. We also compared their performance given a misspecification of the model. Figure 2 displays both correct and misspecified models along with their unstandardized and standardized parameter values. The misspecified model involved two cross loadings and an additional path coefficient, as indicated by dashed arrows in Figure 2.

For both specifications, we considered the same four levels of sample size, at each of which 1,000 random samples were generated from a multivariate normal distribution with zero means and the covariance matrix implied by the unstandardized parameters of the correct model, based on a CSA formulation. Again, we used the maximum likelihood method for CSA, and Mode A and the path weighting scheme for PLSc. We applied all the four methods to estimate the parameters of the correct model, whereas applied only GSCA, GSCA_M_, and CSA to estimate the parameters of the misspecified model because as stated earlier, PLSc was not designed for models involving cross loadings.

Table 2 presents the relative biases (expressed as percentages), standard deviations, and mean square errors of the standardized loadings and path coefficients estimated from the four methods under the correct model specification. GSCA did not encounter non-convergence or the occurrence of improper solutions across all the sample sizes. Conversely, the numbers of the samples omitted with the convergence problems under CSA at the different sample sizes were 72 (*N* = 100), 41 (*N* = 200), 30 (*N* = 500), and 23 (*N* = 1000), whereas those under PLSc were 38 (*N* = 100) and 3 (*N* = 200). One sample was removed under GSCA_M_ only when *N* = 100.

**Table 2:** Relative biases expressed as percentages (RB(%)), standard deviations (SD), and mean square errors (MSE) of standardized loadings and path coefficients obtained from GSCA, GSCA_M_, PLSc, and CSA over different sample sizes.

***N***	**Parameters**		**RB (%)**				**SD**				**MSE**			
			**GSCA**	**GSCA_M_**	**PLSc**	**CSA**	**GSCA**	**GSCA_M_**	**PLSc**	**CSA**	**GSCA**	**GSCA_M_**	**PLSc**	**CSA**
100	Loadings	0.8	4.79	−4.43	−6.25	−5.23	0.03	0.09	0.11	0.08	0.00	0.01	0.01	0.01
		0.7	16.13	0.99	−0.70	−0.04	0.04	0.08	0.11	0.08	0.01	0.01	0.01	0.01
		0.6	31.38	9.97	8.07	8.98	0.04	0.08	0.12	0.08	0.04	0.01	0.02	0.01
		0.7	15.97	2.29	−0.63	0.21	0.04	0.11	0.09	0.08	0.01	0.01	0.01	0.01
		0.6	31.22	10.58	9.72	9.58	0.05	0.11	0.09	0.08	0.04	0.02	0.01	0.01
		0.5	53.36	27.28	24.24	25.08	0.05	0.11	0.10	0.09	0.07	0.03	0.03	0.02
		0.8	4.59	−4.58	−6.19	−5.39	0.03	0.09	0.11	0.08	0.00	0.01	0.01	0.01
		0.7	16.17	1.69	−0.99	0.26	0.04	0.09	0.12	0.08	0.01	0.01	0.01	0.01
		0.4	90.18	52.95	50.88	51.53	0.05	0.09	0.13	0.08	0.13	0.05	0.06	0.05
	Paths	0.6	−26.52	−5.73	1.18	−0.35	0.08	0.10	0.11	0.11	0.03	0.01	0.01	0.01
		0.6	−26.67	−5.40	2.12	0.07	0.08	0.10	0.10	0.10	0.03	0.01	0.01	0.01
200	Loadings	0.8	4.78	−4.96	−5.45	−5.59	0.02	0.06	0.07	0.06	0.00	0.01	0.01	0.01
		0.7	16.11	0.24	−0.51	−0.04	0.03	0.06	0.08	0.05	0.01	0.00	0.01	0.00
		0.6	32.03	10.52	9.15	9.92	0.03	0.06	0.08	0.05	0.04	0.01	0.01	0.01
		0.7	15.87	0.60	−0.11	−0.04	0.03	0.07	0.07	0.06	0.01	0.01	0.00	0.00
		0.6	31.40	10.62	9.38	9.65	0.03	0.07	0.07	0.06	0.04	0.01	0.01	0.01
		0.5	54.06	26.72	25.28	25.80	0.03	0.07	0.07	0.06	0.07	0.02	0.02	0.02
		0.8	4.73	−4.53	−5.56	−5.05	0.02	0.06	0.08	0.05	0.00	0.01	0.01	0.00
		0.7	16.16	0.53	−0.39	−0.11	0.03	0.06	0.09	0.06	0.01	0.00	0.01	0.00
		0.4	90.38	52.38	50.93	51.63	0.04	0.07	0.09	0.06	0.13	0.05	0.05	0.05
	Paths	0.6	−26.65	−2.30	0.72	−0.05	0.06	0.07	0.08	0.08	0.03	0.01	0.01	0.01
		0.6	−27.15	−2.33	1.05	0.25	0.06	0.07	0.07	0.07	0.03	0.01	0.01	0.00
500	Loadings	0.8	4.93	−4.95	−5.10	−5.03	0.01	0.04	0.05	0.03	0.00	0.00	0.00	0.00
		0.7	16.23	0.21	−0.33	−0.10	0.02	0.04	0.05	0.03	0.01	0.00	0.00	0.00
		0.6	32.10	10.07	9.73	9.88	0.02	0.04	0.05	0.03	0.04	0.00	0.01	0.00
		0.7	15.99	0.50	−0.13	0.09	0.02	0.04	0.04	0.03	0.01	0.00	0.00	0.00
		0.6	31.42	9.90	9.53	9.63	0.02	0.04	0.04	0.04	0.04	0.01	0.01	0.00
		0.5	54.10	25.82	25.66	25.68	0.02	0.04	0.04	0.04	0.07	0.02	0.02	0.02
		0.8	4.73	−4.89	−5.25	−5.13	0.01	0.04	0.05	0.03	0.00	0.00	0.00	0.00
		0.7	16.27	0.37	−0.37	0.09	0.02	0.04	0.05	0.03	0.01	0.00	0.00	0.00
		0.4	90.60	51.88	52.13	51.73	0.02	0.04	0.06	0.04	0.13	0.04	0.05	0.04
	Paths	0.6	−27.28	−1.18	−0.07	−0.35	0.04	0.05	0.05	0.05	0.03	0.00	0.00	0.00
		0.6	−27.57	−0.67	0.55	0.22	0.04	0.05	0.05	0.05	0.03	0.00	0.00	0.00
1,000	Loadings	0.8	4.94	−4.95	−5.20	−5.06	0.01	0.03	0.03	0.02	0.00	0.00	0.00	0.00
		0.7	16.24	0.04	−0.16	−0.06	0.01	0.03	0.03	0.02	0.01	0.00	0.00	0.00
		0.6	32.05	9.83	9.83	9.77	0.01	0.03	0.04	0.03	0.04	0.00	0.00	0.00
		0.7	15.94	0.07	−0.07	−0.06	0.01	0.03	0.03	0.02	0.01	0.00	0.00	0.00
		0.6	31.40	9.62	9.53	9.55	0.01	0.03	0.03	0.03	0.04	0.00	0.00	0.00
		0.5	54.06	25.80	25.38	25.52	0.02	0.03	0.03	0.03	0.07	0.02	0.02	0.02
		0.8	4.71	−5.14	−5.06	−5.18	0.01	0.03	0.03	0.02	0.00	0.00	0.00	0.00
		0.7	16.33	0.36	0.07	0.20	0.01	0.03	0.03	0.02	0.01	0.00	0.00	0.00
		0.4	90.73	52.10	51.55	51.88	0.02	0.03	0.04	0.03	0.13	0.04	0.04	0.04
	Paths	0.6	−27.15	−0.32	0.23	0.10	0.03	0.03	0.03	0.03	0.03	0.00	0.00	0.00
		0.6	−27.93	−0.47	0.12	0.07	0.03	0.03	0.03	0.03	0.03	0.00	0.00	0.00

*A factor-based structural equation model with unequal loadings was specified*.

As expected, all the loading estimates from GSCA except those of two high loadings (0.8) were positively biased, whereas all the path coefficient estimates were negatively biased, regardless of the sample sizes. Conversely, overall, GSCA_M_, PLSc, and CSA tended to result in unbiased estimates of a majority of loadings and all path coefficients across the sample sizes. However, the estimates of two low loadings (0.4 or 0.5) under these methods remained similar in magnitude and positively biased even when *N* = 1,000. As in the first simulation study, when *N* = 100, the path coefficient estimates of GSCA_M_ showed larger relative biases than those from PLSc and CSA, although they decreased rapidly with the sample size, approaching zero when *N* = 1,000.

The standard deviations of the estimates from the four methods became smaller with the sample size. GSCA provided smaller standard deviations than the other methods. The mean square errors of all the parameter estimates obtained from GSCA_M_, PLSc, and CSA remained similar in magnitude across the sample sizes and most of them, except for those for the estimates of the two loadings, approached zero when the sample size increased. On the other hand, those from GSCA remained larger and only a few approached zero, although they gradually decreased with the sample size.

Table 3 shows the relative biases (expressed as percentages), standard deviations, and mean square errors of the standardized loadings and path coefficients estimated from GSCA, GSCA_M_, and CSA under the incorrect model specification. CSA suffered severely from non-convergence or convergence to improper solutions across all the sample sizes. The numbers of the samples omitted under CSA were 371 (*N* = 100), 216 (*N* = 200), 131 (*N* = 500), and 100 (*N* = 1,000), whereas those under GSCA_M_ were 54 (*N* = 100), 12 (*N* = 200), 1 (*N* = 500), and 1 (*N* = 1,000). Again, GSCA had no such problems across all the sample sizes.

**Table 3:** Relative biases expressed as percentages (RB(%)), standard deviations (SD), and mean square errors (MSE) of standardized loadings and path coefficients obtained from GSCA, GSCA_M_, PLSc, and CSA over different sample sizes.

***N***	**Parameters**		**RB (%)**			**SD**			**MSE**		
			**GSCA**	**GSCA_M_**	**CSA**	**GSCA**	**GSCA_M_**	**CSA**	**GSCA**	**GSCA_M_**	**CSA**
100	Loadings	0.8	2.90	−4.35	−4.89	0.04	0.08	0.07	0.00	0.01	0.01
		0.7	13.76	0.71	−0.01	0.04	0.08	0.07	0.01	0.01	0.01
		0.6	28.48	10.13	8.87	0.05	0.08	0.08	0.03	0.01	0.01
		0.0	12.89	2.08	1.72	0.10	0.13	0.16	0.03	0.02	0.03
		0.7	2.84	−0.80	−1.73	0.08	0.16	0.14	0.01	0.03	0.02
		0.6	27.92	11.15	11.73	0.05	0.10	0.08	0.03	0.02	0.01
		0.5	49.14	27.64	26.32	0.06	0.11	0.08	0.06	0.03	0.02
		0.0	12.72	2.14	4.01	0.08	0.11	0.15	0.02	0.01	0.02
		0.8	−4.31	−7.20	−9.75	0.06	0.13	0.13	0.01	0.02	0.02
		0.7	15.90	2.26	2.67	0.04	0.09	0.08	0.01	0.01	0.01
		0.4	89.50	53.35	53.88	0.05	0.09	0.09	0.13	0.05	0.05
	Paths	0.6	−5.18	−6.68	−4.50	0.08	0.11	0.12	0.01	0.01	0.01
		0.0	−1.95	3.99	1.48	0.11	0.16	0.19	0.01	0.03	0.04
		0.6	−0.65	−8.78	−6.22	0.11	0.15	0.18	0.01	0.03	0.03
200	Loadings	0.8	2.80	−5.10	−5.66	0.02	0.06	0.05	0.00	0.01	0.00
		0.7	13.73	0.17	−0.40	0.03	0.06	0.05	0.01	0.00	0.00
		0.6	29.08	10.47	9.88	0.03	0.06	0.05	0.03	0.01	0.01
		0.0	13.40	1.08	0.66	0.07	0.08	0.12	0.02	0.01	0.01
		0.7	2.69	−0.53	−0.39	0.05	0.11	0.11	0.00	0.01	0.01
		0.6	27.90	10.60	10.28	0.03	0.07	0.06	0.03	0.01	0.01
		0.5	49.72	26.62	26.28	0.04	0.07	0.06	0.06	0.02	0.02
		0.0	12.38	0.62	0.62	0.05	0.08	0.11	0.02	0.01	0.01
		0.8	−3.60	−5.24	−5.76	0.04	0.10	0.11	0.00	0.01	0.01
		0.7	15.69	0.63	0.63	0.03	0.06	0.06	0.01	0.00	0.00
		0.4	89.35	52.40	51.75	0.04	0.06	0.06	0.13	0.05	0.05
	Paths	0.6	−4.88	−2.32	−1.40	0.06	0.08	0.08	0.00	0.01	0.01
		0.0	−2.21	1.07	0.18	0.08	0.13	0.14	0.01	0.02	0.02
		0.6	−0.38	−3.02	−1.27	0.08	0.12	0.13	0.01	0.01	0.02
500	Loadings	0.8	3.01	−4.98	−4.96	0.02	0.04	0.03	0.00	0.00	0.00
		0.7	13.84	0.19	−0.14	0.02	0.04	0.03	0.01	0.00	0.00
		0.6	29.22	10.07	9.93	0.02	0.04	0.03	0.03	0.00	0.00
		0.0	12.98	0.16	−0.33	0.04	0.05	0.07	0.02	0.00	0.01
		0.7	3.39	0.29	0.53	0.03	0.07	0.07	0.00	0.00	0.00
		0.6	27.85	9.90	9.82	0.02	0.04	0.04	0.03	0.01	0.00
		0.5	49.68	25.82	25.64	0.02	0.04	0.04	0.06	0.02	0.02
		0.0	12.39	0.13	−0.40	0.03	0.05	0.08	0.02	0.00	0.01
		0.8	−3.48	−5.08	−4.63	0.03	0.06	0.07	0.00	0.01	0.01
		0.7	15.77	0.40	0.23	0.02	0.04	0.04	0.01	0.00	0.00
		0.4	89.53	51.95	52.03	0.02	0.04	0.04	0.13	0.04	0.04
	Paths	0.6	−5.12	−1.32	−0.43	0.03	0.05	0.05	0.00	0.00	0.00
		0.0	−2.01	0.39	−0.19	0.05	0.08	0.08	0.00	0.01	0.01
		0.6	−0.68	−0.92	0.28	0.05	0.08	0.08	0.00	0.01	0.01
1,000	Loadings	0.8	3.00	−4.98	−5.04	0.01	0.03	0.02	0.00	0.00	0.00
		0.7	13.87	0.04	−0.07	0.01	0.03	0.02	0.01	0.00	0.00
		0.6	29.17	9.83	9.72	0.01	0.03	0.03	0.03	0.00	0.00
		0.0	13.00	0.09	−0.27	0.03	0.04	0.05	0.02	0.00	0.00
		0.7	3.37	−0.03	0.37	0.02	0.05	0.05	0.00	0.00	0.00
		0.6	27.78	9.65	9.53	0.02	0.03	0.03	0.03	0.00	0.00
		0.5	49.56	25.70	25.54	0.02	0.03	0.03	0.06	0.02	0.02
		0.0	12.45	0.22	−0.07	0.02	0.03	0.06	0.02	0.00	0.00
		0.8	−3.45	−5.30	−4.98	0.02	0.04	0.05	0.00	0.00	0.00
		0.7	15.81	0.36	0.21	0.01	0.03	0.03	0.01	0.00	0.00
		0.4	89.58	52.13	52.03	0.02	0.03	0.03	0.13	0.04	0.04
	Paths	0.6	−4.92	−0.38	0.17	0.02	0.03	0.04	0.00	0.00	0.00
		0.0	−1.83	0.31	0.02	0.03	0.06	0.06	0.00	0.00	0.00
		0.6	−1.12	−0.62	−0.13	0.03	0.06	0.06	0.00	0.00	0.00

*A misspecification of a factor-based structural equation model with unequal loadings was considered*.

Overall, GSCA_M_ and CSA tended to produce unbiased estimates of most of the loadings and all the path coefficients across the sample sizes. However, their estimates of two low loadings (0.4 or 0.5) remained similar in magnitude and positively biased even when *N* = 1,000. GSCA resulted in positively biased loading estimates except those of two high loadings (0.8) regardless of the sample sizes. Conversely, it produced path coefficient estimates with an acceptably small amount of bias, although the amount of bias on average remained unchanged over the sample sizes.

The standard deviations of the estimates from the three methods became smaller with the sample size. Again, GSCA provided smaller standard deviations than the other methods. The mean square errors of all the parameter estimates obtained from GSCA_M_ and CSA were similar in magnitude across the sample sizes and most of them, except for those for the estimates of the two loadings, approached zero when the sample size increased. On the other hand, the mean square errors of the loading estimates from GSCA remained larger and only a few approached zero, although they gradually decreased with the sample size. The mean square errors of the path coefficient estimates from GSCA were comparable to those from GSCA_M_ and CSA across the sample sizes.

To summarize, GSCA_M_ was found to recover the parameters equally well to CSA in both simulation studies that generated data within the factor-analytic framework. When the basic design held for the specified model, PLSc also performed equally well to GSCA_M_ and CSA. In general, GSCA_M_ was less likely to suffer from non-convergence or the occurrence of improper solutions than CSA and PLSc. In particular, CSA tended to suffer from these problems when the sample size was small and/or the model was misspecified, which was consistent with the literature (e.g., Boomsma, 1982, 1985; Anderson and Gerbing, 1984). Conversely, GSCA largely resulted in biased parameter estimates in these simulation studies that were based on the assumption of factor-analytic models.

## An empirical application

**Figure 3:**
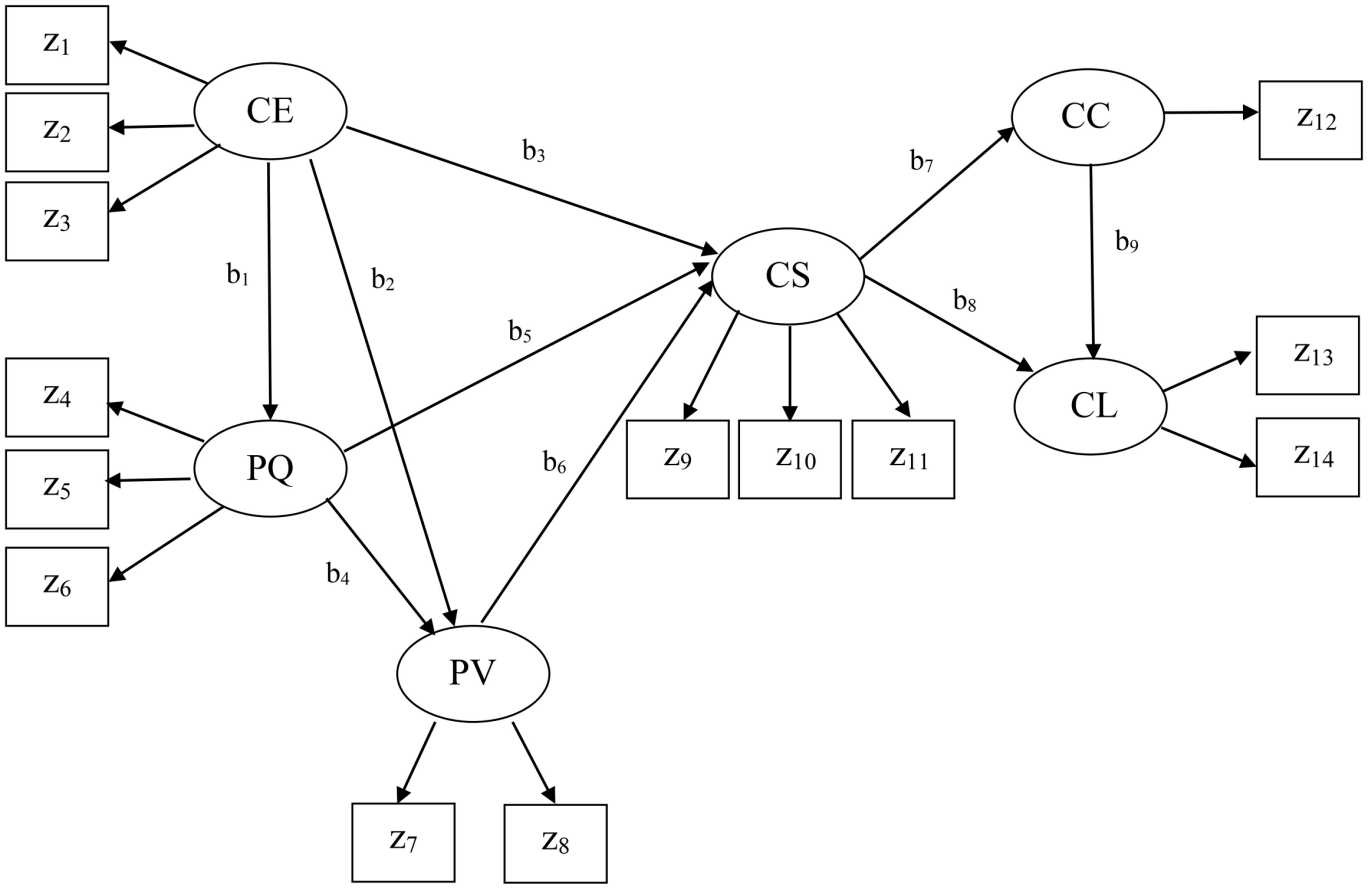
The American customer satisfaction index model. No residual terms are displayed.

The present example came from the American customer satisfaction index (ACSI; Fornell et al., 1996) database. The ACSI has been widely used to assess four different levels of customer satisfaction (national-, sector-, industry-, and company-level) in the United States over the past two decades. This example was company-level data collected in 2002 for 152 companies (*N* = 152). Figure 3 displays the ACSI model. We did not display the residual terms associated with all endogenous variables to make the figure concise. As depicted in Figure 3, the ACSI model contains fourteen indicators: z_1_ = customer expectations about overall quality, z_2_ = customer expectations about reliability, z_3_ = customer expectations about customization, z_4_ = overall quality, z_5_ = reliability, z_6_ = customization, z_7_ = price given quality, z_8_ = quality given price, z_9_ = overall customer satisfaction, z_10_ = confirmation of expectations, z_11_ = distance to ideal product or service, z_12_ = formal or informal complaint behavior, z_13_ = repurchase intention, and z_14_ = price tolerance. The measures and scales of the indicators are described in Fornell et al. (1996). This model also involves six latent variables that underlie the 14 indicators, as follows: CE = customer expectations, PQ = perceived quality, PV = perceived value, CS = customer satisfaction, CC = customer complaints, and CL = customer loyalty. The specified relationships in the ACSI model were well-derived from previous theories, and their detailed conceptual derivations can be found in Fornell et al. (1996).

We applied GSCA_M_, CSA, and PLSc to fit the ACSI model to the data. We used the R packages lavaan (version 0.5-16) (Rosseel, 2012) to apply CSA and wrote MATLAB codes for GSCA_M_ and PLSc. As in the simulation study, we utilized maximum likelihood for CSA, and Mode A and the path weighting scheme for PLSc.

Note that in the ACSI model, only a single indicator (z_12_) loads on customer complaints. As discussed earlier, PLSc was developed based on the basic design requiring at least two indicators per latent variable. When there is only one indicator for a latent variable, PLSc cannot estimate its loading and the path coefficients involving the latent variable because the correction constant for the indicator becomes zero (see Dijkstra and Henseler, 2015). Thus, we used the PLSPM estimate of the component weight for the indicator z_12_, which was equal to one, by fixing the correction constant to one. This was also the case when estimating the path coefficients involving customer complaints (b_7_ and b_9_), indicating that they might be suboptimal estimates of the path coefficients.

Tables 4, 5 provide the estimates of the standardized loadings and path coefficients of the ACSI model obtained from the methods. CSA yielded a number of improper solutions, including the standardized loading and path coefficient estimates greater than one in absolute value. In addition, the loading estimate for z_1_ (customer expectations about overall quality) was almost zero. This was inconsistent with that the indicator is expected to be highly and positively related to customer expectations (Fornell et al., 1996). PLSc also produced improper solutions, although it resulted in fewer than CSA. We have tried different schemes, but continued to have the same problem. Moreover, the positive signs of the path coefficient estimates involving customer complaints (b_7_ and b_9_) were substantively contradictory, suggesting that more satisfied customers tended to complain more frequently (b_7_ = 0.46) and more frequent complainers were likely to be more loyal customers (b_9_ = 0.44). These counterintuitive signs were provided, albeit the signs of all the loading estimates remained positive as expected, indicating that the latent variables were not likely to be sign-reversed.

**Table 4:** The estimates of standardized loadings of the ACSI model obtained from CSA, PLSc, and GSCA_M_.

**Latent**	**Indicator**	**CSA**	**PLSc**	**GSCA_M_**
CE	z_1_	0.00	0.91	0.95
	z_2_	−0.91	0.94	0.97
	z_3_	−0.94	0.96	0.92
PQ	z_4_	0.96	0.98	0.97
	z_5_	0.97	0.97	0.96
	z_6_	0.94	0.93	0.97
PV	z_7_	0.93	0.90	0.96
	z_8_	1.02	1.05	0.99
CS	z_9_	1.00	0.98	0.99
	z_10_	0.98	0.94	0.99
	z_11_	0.90	0.95	0.92
CC	z_12_	1.00	1.00	1.00
CL	z_13_	0.93	0.94	0.96
	z_14_	1.00	1.00	0.99

**Table 5:** The estimates of standardized path coefficients of the ACSI model obtained from CSA, PLSc, and GSCA_M_.

	**CSA**	**PLSc**	**GSCA_M_**
CE → PQ (b_1_)	−0.97	0.95	0.93
CE → PV (b_2_)	1.35	−0.28	−0.11
CE → CS (b_3_)	1.83	−0.21	−0.05
PQ → PV (b_4_)	2.23	1.10	0.93
PQ → CS (b_5_)	2.90	1.01	0.80
PV → CS (b_6_)	−0.13	0.21	0.27
CS → CC (b_7_)	−0.45	0.46	−0.46
CS → CL (b_8_)	0.47	0.51	0.50
CC → CL (b_9_)	−0.47	0.44	−0.45

Conversely, GSCA_M_ resulted in neither improper solutions nor estimates that made little substantive sense. It provided that FIT = 0.85, indicating that the ACSI model accounted for about 85% of the variance of all the variables. Moreover, GSCA_M_ provided that FIT_M_ = 0.98 and FIT_S_ = 0.57. This indicates that the measurement model of the ACSI accounted for about 98% of the variance of the indicators, whereas the structural model explained about 57% of the variance of the latent variables. As also shown in Table 4, all the loading estimates were large and positive. The interpretations of the path coefficient estimates appeared to be generally consistent with those reported in the literature (e.g., Fornell et al., 1996; Anderson and Fornell, 2000). Specifically, customer expectations had a statistically significant impact on perceived quality (b_1_ = 0.93, 95% CI = 0.90 ~ 0.95), but had statistically non-significant effects on perceived value (b_2_ = −0.11, 95% CI = −0.48 ~ 0.22) and customer satisfaction (b_3_ = −0.05, 95% CI = −0.18 ~ 0.05). These non-significant effects were also discussed in previous studies (e.g., Johnson et al., 2001). Perceived quality had statistically significant effects on perceived value (b_4_ = 0.93, 95% CI = 0.54 ~ 1.33) and customer satisfaction (b_5_ = 0.80, 95% CI = 0.66 ~ 0.98). Perceived value had a statistically significant influence on customer satisfaction (b_6_ = 0.27, 95% CI = 0.21 ~ 0.34). Customer satisfaction had statistically significant effects on customer complaints (b_7_ = −0.46, 95% CI = −0.63 ~ −0.30) and customer loyalty (b_8_ = 0.50, 95% CI = 0.39 ~ 0.61). Customer complaints had a statistically significant effect on customer loyalty (b_9_ = −0.45, 95% CI = −0.56 ~ −0.37). We used 100 bootstrap samples for the estimation of the 95% confidence intervals of the GSCA_M_ estimates.

To summarize, in this application, CSA and PLSc yielded improper solutions that were problematic to interpret. The improper solutions may have occurred for reasons. For example, a few latent variables underlie only two indicators each in the ACSI model, the sample size was relatively small, the correlation between customer expectations and perceived quality, which was equivalent to the standardized path coefficient between them (b_1_), was quite large (>|0.90|), or a combination of these issues (e.g., Chen et al., 2001). In addition, both CSA and PLSc provided estimates that were substantively counterintuitive. For the same data, conversely, GSCA_M_ did not result in improper solutions and its estimates were generally consistent with the hypothesized relationships in the literature.

## Concluding remarks

We proposed an extension of GSCA, named GSCA_M_, to explicitly accommodate errors in indicators. As with GSCA, GSCA_M_ can be viewed as a component-based approach to SEM in that it still approximates a latent variable by a component. Unlike GSCA, however, GSCA_M_ considers both common and unique parts of indicators as in factor-based SEM, and estimates a component of indicators with their unique parts excluded. In this way, GSCA_M_ deals with measurement errors in indicators, yielding parameter estimates comparable to those from factor-based SEM. In addition, it does not require a distributional assumption, such as multivariate normality of indicators, for parameter estimation because it estimates parameters via least squares. As a component-based approach, furthermore, it can avoid factor score indeterminacy (e.g., Guttman, 1955; Schönemann and Wang, 1972), enabling to provide unique latent variable scores.

In the simulation studies, GSCA_M_ performed equally well to CSA and PLSc in parameter recovery, when the model was correctly specified to satisfy the basic design assumption. Conversely, when the model was misspecified to contain additional cross loadings and path coefficients, only GSCA_M_ and CSA could be applied to fit the model; and GSCA_M_ tended to recover parameters equally to CSA. In the real data application, GSCA_M_ was the only method that involved no improper solutions.

Although we do not venture into generalizing the results of our analyses, GSCA_M_ may be a promising alternative to CSA, when researchers have difficulty to address such issues as non-convergence or convergence to improper solutions, or are interested in obtaining unique individual latent variable scores for subsequent analyses or modeling of these scores. This can contribute to widening the scope and applicability of GSCA. Nonetheless, it would be fruitful to apply the proposed method to a wide range of real-world problems to investigate its performance more thoroughly.

## Author contributions

HH contributed to conducing all research activities including technical development, empirical analyses, and manuscript writing; YT contributed to technical development and manuscript writing; and KJ contributed to empirical analyses and manuscript writing.

## Conflict of interest statement

The authors declare that the research was conducted in the absence of any commercial or financial relationships that could be construed as a potential conflict of interest.
