# Supplementary material for: Generalized Structured Component Analysis with Uniqueness Terms for Accommodating Measurement Error
Source: Front Psychol. 2017 Dec 6;8:2137. doi: 10.3389/fpsyg.2017.02137 (PMC5723672; doi:10.3389/fpsyg.2017.02137)
Supplement: Supplementary file 1 [file DataSheet1.pdf]

Appendix

The proposed algorithm repeats the following steps until convergence, e.g., until no substantial difference in parameter estimates occurs between iterations.

Step 1: Update $\mathbf{\Gamma}$ (or equivalently $\mathbf{W}$) for fixed $\mathbf{A}$, $\mathbf{U}$, and $\mathbf{D}$. We can re-express (5) as follows.

$$\begin{aligned}\phi &= \mathrm{SS}([\mathbf{Z}, \mathbf{\Gamma}] - \mathbf{\Gamma A} - [\mathbf{UD}, \mathbf{0}]) \\ &= \mathrm{SS}([\mathbf{Z} - \mathbf{UD}, \mathbf{0}] + \mathbf{\Gamma}[\mathbf{0}, \mathbf{I}_P] - \mathbf{\Gamma A}) \\ &= \mathrm{SS}((\mathbf{Z} - \mathbf{UD})[\mathbf{I}_J, \mathbf{0}] - \mathbf{\Gamma}[\mathbf{A} - [\mathbf{0}, \mathbf{I}_P]]) \\ &= \mathrm{SS}((\mathbf{Z} - \mathbf{UD})\mathbf{M} - \mathbf{\Gamma H}),\end{aligned} \tag{A1}$$

where $\mathbf{H} = \mathbf{A} - [\mathbf{0}, \mathbf{I}_P]$, and $\mathbf{M} = [\mathbf{I}_J, \mathbf{0}]$. The least squares estimate of $\mathbf{\Gamma}$ is obtained by

$$\hat{\mathbf{\Gamma}} = (\mathbf{Z} - \mathbf{UD})\mathbf{MH}'(\mathbf{HH}')^{-1}, \tag{A2}$$

indicating $\hat{\mathbf{W}} = \mathbf{MH}'(\mathbf{HH}')^{-1}$. Each latent variable is updated by $\hat{\boldsymbol{\gamma}}_p = \mathbf{Z}_p^* \mathbf{w}_p$, where $\mathbf{Z}_p^*$ includes the columns of $\mathbf{Z} - \mathbf{UD}$ corresponding to a set of indicators associated with the pth latent variable, and $\mathbf{w}_p$ contains the elements of the pth column of $\mathbf{W}$ assigned to $\mathbf{Z}_p^*$. Subsequently, it is normalized to satisfy $\hat{\boldsymbol{\gamma}}_p{}'\hat{\boldsymbol{\gamma}}_p = 1$. This can be done by dividing $\mathbf{w}_p$ by $\sqrt{\mathbf{w}_p{}'\mathbf{Z}_p^*{}'\mathbf{Z}_p^*\mathbf{w}_p}$.

Step 2: Update $\mathbf{A}$ for fixed $\mathbf{\Gamma}$, $\mathbf{U}$, and $\mathbf{D}$. This is equivalent to minimizing

$$\begin{aligned}\phi &= \mathrm{SS}(\mathbf{\Psi} - \mathbf{S} - \mathbf{\Gamma A}) \\ &= \mathrm{SS}(\mathrm{vec}(\mathbf{\Psi}^*) - (\mathbf{I}_{J+P} \otimes \mathbf{\Gamma})\mathrm{vec}(\mathbf{A})),\end{aligned} \tag{A3}$$

where $\mathbf{\Psi}^* = \mathbf{\Psi} - \mathbf{S}$. Let $\mathbf{a}$ denote the vector of free elements in vec($\mathbf{A}$). Let $\mathbf{\Phi}$ denote the matrix of the columns of $\mathbf{I} \otimes \mathbf{\Gamma}$ corresponding to the free elements in vec($\mathbf{A}$). Then, the least squares estimate of $\mathbf{a}$ is obtained by

$$\hat{\mathbf{a}} = (\mathbf{\Phi}'\mathbf{\Phi})^{-1}\mathbf{\Phi}'\mathrm{vec}(\mathbf{\Psi}^*). \tag{A4}$$

The updated $\mathbf{A}$ is reconstructed from $\hat{\mathbf{a}}$.

Step 3: Update $\mathbf{U}$ for fixed $\mathbf{\Gamma}$, $\mathbf{A}$, and $\mathbf{D}$. This is equivalent to minimizing

$$\begin{aligned}\phi &= \mathrm{SS}(\mathbf{\Psi} - \mathbf{\Gamma A} - \mathbf{S}) \\ &= \mathrm{SS}([\mathbf{Z} - \mathbf{UD}, (\mathbf{Z} - \mathbf{UD})\mathbf{W}] - (\mathbf{Z} - \mathbf{UD})\mathbf{WA}) \\ &= \mathrm{SS}((\mathbf{Z} - \mathbf{UD})(\mathbf{V} - \mathbf{WA}))\end{aligned} \tag{A5}$$

with respect to **U**, subject to $\mathbf{U}'\mathbf{\Gamma} = \mathbf{0}$ and $\mathbf{U}'\mathbf{U} = \mathbf{I}_J$, where **V** = [**I**, **W**]. We can update **U** based on Trendafilov et al.'s (2013) procedure. Let **Γ** = **QR** = $[\tilde{\mathbf{\Gamma}}, \mathbf{\Gamma}_\perp]\begin{bmatrix}\mathbf{I} \\ \mathbf{O}\end{bmatrix}$ denote the QR decomposition of **Γ**, where $\mathbf{\Gamma}_\perp$ is an *N* by *N* – *P* orthonormal basis matrix of the null space of **Γ**. Minimizing (A5) is equivalent to minimizing

$$\begin{aligned}\phi^* &= \mathrm{SS}(\mathbf{Q}'(\mathbf{Z} - \mathbf{UD})(\mathbf{V} - \mathbf{WA})) \\ &= \mathrm{SS}\left(\begin{bmatrix}\tilde{\mathbf{\Gamma}}'(\mathbf{Z} - \mathbf{UD})(\mathbf{V} - \mathbf{WA}) \\ \mathbf{\Gamma}_\perp'(\mathbf{Z} - \mathbf{UD})(\mathbf{V} - \mathbf{WA})\end{bmatrix}\right) \\ &= \mathrm{SS}\left(\begin{bmatrix}\tilde{\mathbf{\Gamma}}'\mathbf{Z}(\mathbf{V} - \mathbf{WA}) \\ \mathbf{\Gamma}_\perp'(\mathbf{Z} - \mathbf{UD})(\mathbf{V} - \mathbf{WA})\end{bmatrix}\right) \\ &= \mathrm{tr}(\mathbf{V}'\mathbf{Z}'\tilde{\mathbf{\Gamma}}\tilde{\mathbf{\Gamma}}'\mathbf{ZV}) + \mathrm{tr}(\mathbf{Z}'\mathbf{\Gamma}_\perp\mathbf{\Gamma}_\perp'\mathbf{Z}) + \mathrm{tr}(\mathbf{A}'\mathbf{W}'\mathbf{Z}'\tilde{\mathbf{\Gamma}}\tilde{\mathbf{\Gamma}}'\mathbf{ZWA}) + \mathrm{tr}(\mathbf{D}^2) \\ &\quad - 2\mathrm{tr}(\mathbf{V}'\mathbf{Z}'\tilde{\mathbf{\Gamma}}\tilde{\mathbf{\Gamma}}'\mathbf{ZWA}) - 2\mathrm{tr}(\mathbf{U}'\mathbf{\Gamma}_\perp\mathbf{\Gamma}_\perp'\mathbf{ZD}).\end{aligned} \tag{A6}$$

Let us reparametrize **U** as $\mathbf{U} = \mathbf{\Gamma}_\perp\tilde{\mathbf{U}}$, where $\tilde{\mathbf{U}}$ is an *N* – *P* by *J* orthonormal matrix with $\tilde{\mathbf{U}}'\tilde{\mathbf{U}} = \mathbf{I}_J$, thereby keeping the constraint $\mathbf{U}'\mathbf{U} = \mathbf{I}_J$ satisfied. Then, updating **U** reduces to updating $\tilde{\mathbf{U}}$ by maximizing $\mathrm{tr}(\mathbf{U}'\mathbf{\Gamma}_\perp\mathbf{\Gamma}_\perp'\mathbf{ZD}) = \mathrm{tr}(\tilde{\mathbf{U}}'\mathbf{\Gamma}_\perp'\mathbf{ZD})$ in (A6), subject to $\tilde{\mathbf{U}}'\tilde{\mathbf{U}} = \mathbf{I}_J$. This problem can be solved as follows: Let SVD($\mathbf{\Gamma}_\perp'\mathbf{ZD}$) = **PΔT**′. Then, $\hat{\tilde{\mathbf{U}}} = \mathbf{PT}'$ (e.g., ten Berge, 1993, p. 34). Finally, **U** is updated by $\hat{\mathbf{U}} = \mathbf{\Gamma}_\perp\hat{\tilde{\mathbf{U}}}$.

<u>Step 4:</u> Update **D** for fixed **Γ**, **A**, and **U**. Minimizing (5) with respect to **D** is equivalent to minimizing

$$\phi = \mathrm{SS}(\mathbf{Z}(\mathbf{V} - \mathbf{WA}) - \mathbf{UD}(\mathbf{V} - \mathbf{WA})). \tag{A7}$$

Then, the least squares estimate of **D** is obtained by

$$\hat{\mathbf{D}} = \mathrm{diag}(\mathbf{U}'\mathbf{Z}). \tag{A8}$$
